# Increased Mortality and Complication Rates in Weekend Admissions for Acute Decompensated Heart Failure: A Five-Year National Study

**DOI:** 10.3390/jcm15062097

**Published:** 2026-03-10

**Authors:** Hadi Itani, Mohammad Ennab, Mohamad Bahij Moumneh, Elie Bou Sanayeh, Elie Moussa, Bahy Abofrekha, Ahmed Zayed, Omar Khayat, Martin Amor

**Affiliations:** 1 Department of Internal Medicine, Staten Island University Hospital, Northwell Health, New Hyde Park, NY 10305, USAmmoumneh@northwell.edu (M.B.M.);; 2 Department of Cardiology, Staten Island University Hospital, Northwell Health, New Hyde Park, NY 10305, USA

**Keywords:** acute decompensated heart failure, acute kidney injury, respiratory failure, cardiac arrest, mortality, weekend, weekday

## Abstract

**Background/Objectives:** The “weekend effect,” characterized by increased mortality and complication rates for weekend hospital admissions, is well documented in myocardial infarction and stroke but has been less thoroughly investigated in acute decompensated heart failure (ADHF). This study evaluates the weekend effect in ADHF using a national cohort. **Methods:** A retrospective cohort study was conducted using the 2016–2020 Nationwide Inpatient Sample (NIS). Adult ADHF admissions were identified by ICD-10 codes and classified as weekend or weekday admissions. Over 30 variables, including age, sex, and comorbidities, were analyzed. Propensity score matching (1:1) yielded 489,204 patients per group. Univariate and multivariate logistic regression models were used to assess outcomes, adjusting for key covariates. **Results:** Of 2,131,915 ADHF hospitalizations, 501,076 (23.5%) occurred on weekends. The cohort was 48% female, with a mean age of 72 years (SD ± 12.3). After 1:1 matching, weekend admissions had higher odds of cardiac arrest (aOR: 1.10; 95% CI: 1.06–1.13, *p* < 0.001), inpatient mortality (aOR: 1.07; 95% CI: 1.05–1.09, *p* < 0.001), acute kidney injury (AKI; aOR: 1.07; 95% CI: 1.06–1.08, *p* < 0.001), and acute respiratory failure (ARF; aOR: 1.28; 95% CI: 1.27–1.30, *p* < 0.001). No significant differences were observed in mechanical circulatory support (MCS) use or length of stay. **Conclusions:** Weekend ADHF admissions were associated with a higher risk of mortality and complications, which may be attributable to reduced specialist availability or delayed diagnostics. These findings underscore the need for standardized ADHF protocols to ensure equitable care throughout the week.

## 1. Introduction

Heart failure (HF) is a clinical syndrome characterized by functional and/or structural abnormalities that lead to reduced cardiac output and/or elevated intracardiac pressures [1]. It affects around 37–64 million individuals worldwide, corresponding to a prevalence of 800–900 cases per 100,000 individuals (around 1–3% in the adult population) [1,2]. In the United States, an estimated 5.7 million adults were diagnosed with heart failure in 2011, with nearly 870,000 incident HF cases diagnosed annually [1,2].

Acute decompensated heart failure (ADHF), the acute worsening of chronic HF with congestion and hemodynamic compromise, is the third most common cause of unscheduled hospital admission, excluding pregnancy and neonatal cases [3]. It is associated with substantial morbidity, mortality, and healthcare expenditures. In the United States, HF-related healthcare costs are projected to increase from $20.9 billion in 2012 to $53.1 billion by 2030 [1,4].

Despite advances in guideline-directed medical therapy (GDMT) and device management, post-discharge prognosis for HF remains poor. Approximately 24% of patients are readmitted within 30 days, and nearly 35% die within one year of diagnosis [2,4].

Given the acuity of ADHF, rapid diagnosis and prompt initiation of decongestive therapies are essential for optimal clinical outcomes. However, hospital resources and operations often differ during the night and on weekends. This variation has led to the hypothesis of a “weekend effect,” in which patients admitted on weekends experience worse clinical outcomes than those admitted on weekdays [5]. Similar effects have been observed in other urgent conditions, such as acute coronary syndrome [6].

Proposed explanations for this variation include reduced nurse-to-patient ratios, limited access to subspecialists, and delayed access to critical diagnostics or procedures during off-hours [6].

The presence of a weekend effect in ADHF hospitalizations is debated in the literature. Some large registry analyses, including the “Get With The Guidelines-Heart Failure” (GWTG-HF) registry, show higher mortality for weekend admissions [7]. In contrast, studies using the OPTIMIZE-HF registry and the Atherosclerosis Risk in Communities (ARIC) surveillance found no significant difference in mortality by admission day [8].

These conflicting findings may be attributable to differences in study design, such as varying definitions of ‘weekend,’ heterogeneity in hospital types, and challenges in controlling for illness severity. Clarifying this relationship is essential for quality improvement and resource allocation. This study uses a large national cohort to examine the impact of admission timing on outcomes in ADHF. The primary objective is to determine whether weekend admissions for acute decompensated heart failure are associated with higher in-hospital mortality and complication rates relative to weekday admissions.

## 2. Materials and Methods

### 2.1. Data Source

A retrospective cohort study was conducted using the NIS database from 2016 to 2020. The NIS is the largest publicly available, all-payer inpatient database in the United States and is maintained by the Healthcare Cost and Utilization Project (HCUP). It includes a 20% stratified sample of hospital discharges, with discharge-level weights that enable national estimates of hospitalizations and outcomes. The NIS contains information on patient demographics, diagnoses, procedures, comorbidities, hospital characteristics, and discharge disposition [9,10,11].

### 2.2. Study Population

Adult hospitalizations (aged ≥18 years) with a principal or secondary diagnosis of acute decompensated heart failure were identified using International Classification of Diseases, Tenth Revision, Clinical Modification (ICD-10-CM) codes consistent with prior NIS-based HF studies (I50.21, I50.23, I50.31, I50.33, I50.41, I50.43, I50.811, I50.813). The exposure of interest was weekend versus weekday admission. Weekend admissions were defined as those occurring on Saturday or Sunday; all others were classified as weekday admissions.

### 2.3. Variable Definitions and Covariates

Demographic variables included age, sex, and race/ethnicity (categorized as White, Black, Hispanic, Asian/Pacific Islander, Native American/Other, and Unknown). Socioeconomic variables included primary expected payer (Medicare, Medicaid, private insurance, self-pay, no charge/charity, and other).

Comorbidities and clinical history were identified using ICD-10-CM codes (Supplementary Table S1). Key conditions included hypertension, diabetes mellitus, hyperlipidemia, hyperthyroidism, hypothyroidism, peripheral artery disease, coronary artery disease, atrial fibrillation/flutter, chronic obstructive pulmonary disease (COPD), obesity, prior stroke, chronic kidney disease (CKD) stages 1–5 (without dialysis), and end-stage renal disease (ESRD) or dialysis dependence. Hospital-level characteristics included U.S. Census region (e.g., New England, Middle Atlantic), teaching status (urban teaching, urban non-teaching, rural), and bedsize categories, as defined by HCUP.

### 2.4. Outcomes

The primary outcome was in-hospital all-cause mortality.

Secondary clinical outcomes included cardiac arrest, development of ARFARF (hypoxic, hypercapnic, or mixed), ARF requiring invasive mechanical ventilation, acute kidney injury (AKI), initiation of hemodialysis during the index hospitalization, cardiogenic shock, and use of mechanical circulatory support, including intra-aortic balloon pump (IABP), Impella, and extracorporeal membrane oxygenation (ECMO).

Secondary resource-utilization outcomes included length of stay, total hospital charges, and discharge disposition (home, short-term hospital, other facility, home health care, against medical advice, in-hospital death, or unknown).

### 2.5. Propensity Score Matching

To reduce confounding due to baseline differences between patients admitted on weekdays and those admitted on weekends, propensity score matching (PSM) was performed. A logistic regression model was used to estimate the probability of weekend admission based on patient demographics (age, sex, race/ethnicity, payer, income quartile), comorbidities (including hypertension, diabetes mellitus, hyperlipidemia, hypothyroidism, hyperthyroidism, peripheral artery disease, coronary artery disease, atrial fibrillation/flutter, COPD, obesity, stroke, CKD, ESRD), and hospital characteristics (region, location, and teaching status).

Patients admitted on weekends were matched 1:1 to those admitted on weekdays using nearest-neighbor matching without replacement. This procedure yielded 489,204 matched hospitalizations in each group. Covariate balance between weekday and weekend groups after matching was assessed by applying standardized mean differences and compared descriptively.

### 2.6. Statistical Analysis

Survey weights were applied in accordance with HCUP recommendations when generating national estimates to obtain design-based national estimates [9].

Descriptive statistics were used to summarize baseline characteristics before and after matching. Categorical variables were compared using chi-square tests, and continuous variables using independent *t*-tests.

In the matched cohort, model-based analysis in the form of univariate and multivariable logistic regression was performed to estimate the association between weekend admission and each binary outcome (e.g., mortality, cardiac arrest, AKI, ARF), with results reported as adjusted odds ratios (aORs) and 95% confidence intervals (CIs). Models adjusted for residual imbalances in demographics, comorbidities, and hospital characteristics after matching. For LOS and total hospital charges (non-adjusted to inflation), linear regression or generalized linear models with appropriate distributions and link functions were used. A two-sided *p*-value < 0.05 was considered statistically significant. Analyses were performed using Stata 18 (StataCorp, College Station, TX, USA).

### 2.7. Ethical Considerations

The NIS contains de-identified discharge-level data. In accordance with institutional policy and federal regulations, this study was considered exempt from institutional review board review and informed consent requirements.

## 3. Results

### 3.1. Study Cohort and Baseline Characteristics

From 2016 to 2020, a total of 2,131,915 hospitalizations for acute decompensated heart failure were identified in the NIS. Of these, 501,076 (23.5%) occurred on weekends and 1,630,839 (76.5%) on weekdays. The overall cohort had a mean age of 72 years (SD ± 12.3), and approximately 48% were female (Table 1).

After 1:1 propensity score matching, 489,204 weekday and 489,204 weekend admissions were included in the analytic cohort. Baseline characteristics of the matched cohort are presented in Table 2. By design, the demographic and clinical profiles were well-balanced between groups. The mean age was similar for weekday and weekend admissions (71.85 vs. 71.69 years, *p* = 0.34), and the proportions of female patients were comparable (48.89% vs. 48.74%, *p* = 0.129). The prevalence of key comorbidities, including hypertension, diabetes mellitus, hyperlipidemia, coronary artery disease, atrial fibrillation/flutter, COPD, obesity, CKD, and ESRD, did not differ meaningfully between groups.

Racial and ethnic distributions were similar, with white patients comprising approximately two-thirds of admissions (69.04% on weekdays vs. 68.41% on weekends), followed by African American (18.08% vs. 18.24%) and Hispanic (7.98% vs. 8.16%) patients. Primary payers were predominantly Medicare (>70% in both groups), with smaller proportions of Medicaid, private insurance, and self-pay patients. Hospital region and teaching status were also comparable across weekday and weekend admissions, with most hospitalizations occurring in urban teaching hospitals.

### 3.2. Clinical Outcomes

In the propensity-matched cohort, weekend admission was associated with higher rates of major in-hospital complications and mortality compared with weekday admission (Table 3).

The in-hospital mortality rate was 5.45% for weekend admissions versus 5.02% for weekday admissions (26,645 vs. 24,546 deaths; *p* < 0.001), corresponding to an adjusted odds ratio of 1.07 (95% CI: 1.05–1.09). Similarly, the incidence of cardiac arrest was higher among weekend admissions (1.84% vs. 1.60%; 8980 vs. 7822 events; *p* < 0.001), with an adjusted odds ratio of 1.10 (95% CI: 1.06–1.13).

ARFARF occurred in 26.23% of weekend admissions compared with 21.59% of weekday admissions (128,295 vs. 105,603 events; *p* < 0.001). This translated into a 28% higher adjusted odds of ARF for patients admitted on weekends (aOR 1.28; 95% CI: 1.27–1.30). The need for invasive mechanical ventilation among those with ARF was similar between groups (3.36% on weekends vs. 3.31% on weekdays; *p* = 0.161), suggesting that the increased ARF incidence did not fully translate into differences in ventilatory support rates.

AKI was also more frequent among weekend admissions (38.17% vs. 36.37%; 186,732 vs. 177,900 events; *p* < 0.001), with an adjusted odds ratio of 1.07 (95% CI: 1.06–1.08). In contrast, initiation of hemodialysis during hospitalization did not significantly differ between weekend and weekday admissions (6.05% vs. 6.08%; *p* = 0.585). Cardiogenic shock occurred slightly more often in weekend admissions (3.93% vs. 3.68%; 19,255 vs. 17,987 events; *p* < 0.001), consistent with a modestly higher burden of hemodynamic instability.

The overall use of MCS (IABP, Impella, or ECMO) was low and showed no clinically relevant difference between groups in absolute terms. Among matched admissions, the vast majority did not receive MCS (98.83% on weekends vs. 98.73% on weekdays), and the low absolute rates of device use were not directionally consistent with a large weekend effect. Table 4 displays the multivariate analysis of developing complications among those with decompensated heart failure admitted on a weekend compared to weekdays.

### 3.3. Resource Employment

Length of stay was similar for weekday and weekend admissions (mean 6.89 vs. 6.77 days; *p* = 0.07), indicating that the excess mortality and complications associated with weekend admission did not translate into longer hospitalizations among survivors.

Total hospital charges, however, were higher for weekend admissions ($82,017 ± $149,492; *p* < 0.001) compared with weekend admissions (mean $78,913 ± $141,298). This pattern likely reflects greater use of diagnostic and procedural interventions during weekday hospitalizations. Discharge disposition was broadly similar between groups, with comparable proportions of patients discharged home, transferred to short-term hospitals or other facilities, receiving home health care, or leaving against medical advice. As expected, in-hospital death was more frequent among weekend admissions, consistent with the observed mortality difference.

## 4. Discussion

In this large, nationally representative cohort of patients hospitalized with acute decompensated heart failure, weekend admission was associated with higher in-hospital mortality and a greater burden of major complications compared with weekday admission, even after rigorous propensity score matching and multivariable adjustment. Weekend admissions had 7% higher adjusted odds of in-hospital death and a 10% higher adjusted odds of cardiac arrest, along with significantly higher odds of ARF and AKI. This evidence is directionally consistent with prior work describing a “weekend effect” across a broad range of acute medical and surgical conditions, with pooled estimates showing roughly a 7–20% relative increase in mortality for weekend versus weekday admissions [10,11,12,13].

### 4.1. Interpretation of Main Findings

The present analysis shows a modest but clinically meaningful weekend effect in contemporary ADHF care. Given the high incidence of ADHF hospitalizations, even a 7–10% relative increase in mortality and complications translates into a substantial excess number of adverse events at the population level. The association persisted after extensive adjustment for demographics, comorbidities, and hospital-level factors, consistent with meta-analytic data showing that a weekend effect persists even after adjustment for severity and case-mix. This suggests that residual differences in illness severity alone are unlikely to justify the observed disparities fully [10,12].

The increased rates of ARF and AKI among weekend admissions indicate possible care-process mechanisms. Prior syntheses of the weekend effect have stressed the dual contributions of patient factors (“sicker weekend patient” hypothesis) and system factors (reduced staffing, slower access to diagnostics and procedures) [10]. In ADHF specifically, delays in achieving adequate decongestion, less frequent titration of diuretics and vasoactive agents, slower escalation to noninvasive or invasive ventilatory support, and later nephrology involvement may all contribute to progressive respiratory and renal compromise. The modestly higher frequency of cardiogenic shock on weekends in the current study mirrors patterns seen in off-hours admissions for acute myocardial infarction, where delays in reperfusion and reduced catheterization-laboratory availability have been implicated in excess mortality and complications [10]. By contrast, MCS was used in a small minority of patients in both groups, and although statistical significance was reached, the absolute rates were below 0.1%, implying that the difference is driven mainly by common care processes rather than disparities [12].

Importantly, the length of stay was similar between weekend and weekday admissions, whereas hospital charges were higher for weekday admissions. General weekend-effect meta-analyses and health policy analyses have noted that weekday care often entails more diagnostic and procedural activity. In contrast, weekend care may be characterized by relative conservatism in interventions and less intensive use of resources, even when outcomes are worse [10]. In this context, the current data suggest that the weekend effect in ADHF is driven less by prolonged hospitalizations and more by early deterioration and in-hospital death. This raises concern that lower spending on weekends may partially reflect delayed or reduced use of clinically indicated diagnostics and interventions, in line with prior “weekend effect” studies in stroke and acute coronary syndrome, where patients admitted on a weekend received fewer or later interventions and developed worse outcomes [13].

### 4.2. Comparison Relative to Prior Studies

Evidence regarding the weekend effect in HF has been mixed. Horwich et al., using the Get With The Guidelines–Heart Failure (GWTG-HF) registry of 81,810 hospitalizations (mean age 72 ± 14 years, 50% male), observed that weekend admissions and discharges were associated with differences in quality metrics and outcomes in HF (e.g., longer hospitalization), raising concerns about off-hours vulnerability [14]. Additionally, weekend HF admission was associated with higher risk-adjusted odds of in-hospital death (aOR 1.13, 95% CI 1.02–1.27, *p* = 0.03) [14]. Additionally, weekend HF admission was associated with higher risk-adjusted odds of in-hospital death (aOR 1.13, 95% CI 1.02–1.27, *p* = 0.03) [14]. Similarly, the ARIC surveillance study, among 39,699 weighted hospitalizations (mean age 76, 48% male), noted that in-hospital mortality doubled for weekend admissions (aOR 2.37, 95% CI 1.93–2.91; *p* < 0.001) [3]. In contrast, analyses from OPTIMIZE-HF (N = 48,612 patients, mean age 73 ± 14 years, 52% female) found no significant differences in short-term mortality by day of admission after risk adjustment (*p* = 0.19), suggesting that a uniform weekend effect in HF is not universal [8,15]. Outside the U.S., the Japanese JCARE-CARD registry (N = 1620, mean age 70.7 years, 59.4% male) similarly reported no independent association between weekend admission and in-hospital death in patients admitted with worsening HF after adjustment for covariates (*p* = 0.136) [16].

More recent multinational data have shifted attention from calendar days to time of day and working vs. non-working hours. An analysis from the REPORT-HF registry (N = 18,553, 61% male) found that patients admitted during non-working hours had similar in-hospital mortality but higher 1-year mortality than those admitted during standard working hours (*p* = 0.76 and 0.006, respectively), suggesting that off-hours admission may affect downstream trajectories even when early mortality is comparable [17]. However, the magnitude and consistency of these effects vary across conditions and datasets. This is consistent with broader work from England and other systems, indicating that both weekend and nighttime admissions can be associated with increased adjusted mortality [18,19,20]. In the National Health Service study (N = 4,640,516, 48% male), Mohammed et al. demonstrated that patients admitted on weekends, whether on an elective or emergency basis, had increased risk of death (elective OR 1.32, 95% CI 1.23–1.41; emergency OR 1.09, 95% CI 1.05–1.13; all *p* < 0.001) compared to weekday admissions [18]. Furthermore, Han et al. demonstrated that, among 246,350 emergency spells, the highest mortality risk was for weekend night-time admissions as well (aOR 1.14, 95% CI 1.08–1.21, *p* = 0.003) [19]. However, the magnitude and consistency of these effects vary across conditions and datasets.

The current study expands this literature in several ways. First, it uses a large, nationally representative sample of U.S. hospitalizations with contemporary ICD-10 coding, including both teaching and non-teaching hospitals across all regions. Second, through employing 1:1 propensity score matching to balance demographics, comorbidities, and hospital characteristics, the analysis reduces confounding by case-mix that has complicated prior observational work. Third, it evaluates a broad spectrum of clinically relevant complications in addition to mortality, demonstrating that weekend admission is associated not only with higher death rates but also with higher odds of ARFARF, AKI, cardiogenic shock, and cardiac arrest, trends consistent with off-hours analyses in other cardiovascular emergencies such as NSTEMI and acute aortic syndromes.

The more pronounced weekend effect on respiratory and renal complications is especially significant. Systematic reviews of the weekend effect have highlighted that processes of care (e.g., timeliness of investigations, adherence to guidelines, and swift escalation to higher levels of care) likely mediate part of the observed mortality gap. In HF, where dynamic volume status and renal function are central to outcomes, small delays in diuretic intensification, ventilatory support, or hemodynamic assessment on weekends may excessively translate into ARF and AKI [20,21].

### 4.3. Potential Mechanisms Underlying the Weekend Effect

First, staffing and expertise differ between weekdays and weekends. Large, policy and evidence-based analyses in the UK and elsewhere have demonstrated that specialist physician coverage and multidisciplinary staffing are typically lower on weekends, even in systems actively implementing 7-day services [21]. These reductions have been temporally associated with higher adjusted mortality for weekend admissions across multiple conditions [22]. In ADHF, fewer in-house cardiologists or HF specialists, higher nurse–patient ratios, and reduced access to respiratory therapists or nephrologists on weekends could delay recognition of deterioration and limit initiation of advanced therapies.

Second, access to diagnostics and procedures may be constrained on weekends. Prior studies of the weekend effect in cardiology and hospital medicine have documented reduced use or longer delays for essential diagnostic and treatment interventions on weekends compared with weekdays, despite similar clinical indications [11,23]. For HF, limited off-hours access to echocardiography, right-heart catheterization, or high-level imaging may impede precise hemodynamic assessment and preload and afterload optimization, causing worsening congestion, respiratory failure, and renal dysfunction.

Third, care coordination and transitions may be less robust during weekends. Studies of HF and other chronic conditions point out the importance of multidisciplinary discharge planning, medication reconciliation, and early post-discharge follow-up in reducing readmissions and improving outcomes. On weekends, reduced availability of case managers, pharmacists, and allied health professionals may impact not only discharge processes, but also in-hospital titration of GDMT and consultation coordination. Even if these processes primarily influence post-discharge trajectories, disruptions early in the hospitalization can still affect in-hospital clinical stability and complication risk [11,13].

Finally, system-level pressures and bed capacity may shape differences in the escalation of care. Analyses of weekend and off-hours admissions across conditions have identified interactions among elective procedural schedules, ICU bed availability, and triage thresholds for higher-acuity care. HF patients admitted during busy weekend periods may experience delays in transfer to monitored units or intensive care, which may explain some of the excess cardiogenic shock and cardiac arrest observed in this cohort [11,24,25].

### 4.4. Clinical and Health System Implications

The higher mortality and complication rates observed among weekend ADHF admissions have actionable implications. First, the results support calls for standardized, protocolized HF pathways explicitly designed for 7-day implementation, including early risk stratification, standardized orders for laboratory and imaging evaluation, and algorithmic titration of diuretics and vasodilators. Evidence from other acute cardiovascular syndromes suggests that well-implemented, around-the-clock pathways can attenuate or eliminate weekend mortality gaps.

Second, these data support efforts to optimize weekend staffing models, particularly in hospitals with a high HF burden. The meta-analytic and policy literature indicate that simple increases in consultant hours alone may not completely resolve the weekend effect, but assuring a minimal level of cardiology/HF expertise, adequate nurse staffing, and ready access to respiratory and nephrology services appears essential [11,13].

Third, the study offers empirical justification for investment in 7-day services and infrastructure. Systematic reviews and national policy analyses from the UK and other health systems have highlighted that the weekend effect is multifactorial, involving staffing, process reliability, and access to diagnostics and procedures [24]. For ADHF, extending the availability of echocardiography, noninvasive ventilation protocols, and step-down or intermediate-care beds past standard weekday hours may be a high-yield strategy to reduce early decompensation and death.

### 4.5. Strengths and Limitations

Strengths of this study include the use of a large, nationally representative NIS cohort, contemporary ICD-10 coding, and rigorous propensity score matching to address baseline imbalances. These design features align with methodological recommendations from recent weekend-effect meta-analyses and policy reviews that emphasize strong risk adjustment and the inclusion of system-level covariates. Evaluating multiple clinically meaningful complications, in addition to mortality, gives a more detailed picture of how weekend admission shapes the in-hospital course of ADHF.

Several limitations warrant consideration. First, as an administrative database, the NIS is subject to potential misclassification of ADHF and comorbidities based on ICD-10 codes and lacks granular clinical data, such as natriuretic peptides, ejection fraction, and hemodynamic parameters. Second, individual-level treatment details, including specific HF therapies and their timing, are unavailable, limiting inferences about process-of-care mediators. Third, repeat hospitalizations by the same patient cannot be identified. Fourth, although propensity score matching and multivariable adjustment were applied, residual confounding from unmeasured factors (e.g., as frailty, code status, functional status, social support) remains possible, as highlighted in prior weekend-effect syntheses. Additionally, the absence of granular clinical data, such as admission vital signs, laboratory results (e.g., blood pressure, eGFR, lactate, B-type Natriuretic Peptide [BNP] levels), and left ventricular ejection fraction (LVEF), could introduce residual confounding, even following propensity matching. Finally, the observational design precludes causal inference; the associations between weekend admission and outcomes should therefore be viewed as hypothesis-generating and complementary to more granular registry and quality-improvement studies.

## 5. Conclusions

In a large, nationally representative cohort of adults hospitalized with acute decompensated heart failure, weekend admission was associated with higher in-hospital mortality and increased odds of major complications, including cardiac arrest, ARF, and AKI, compared with weekday admission. These results are consistent with the broader literature documenting a weekend effect across multiple conditions and settings, and with contemporary HF data highlighting vulnerability during off-hours. Collectively, the evidence suggests that time-based variations in staffing, resource readiness, and reliability of care processes add to outcome disparities in ADHF. Applying standardized HF pathways, strengthening weekend coverage, and expanding 7-day diagnostic and critical-care services may help mitigate the weekend effect and promote more unbiased outcomes for patients hospitalized with ADHF, regardless of the day of admission.

## Figures and Tables

**Table 1:** Baseline Demographics of Patients Admitted with Acute Decompensated Heart Failure on Weekday vs. Weekend (before matching).

Variable	Weekday (*n* = 1,631,992)	Weekend (*n* = 501,076)	*p*-Value
Age (years)	Mean = 71.51, SD = 14.09	Mean = 71.69, SD = 14.14	<0.001
	(*n*, %)	(*n*, %)	
Female	777,099 (47.62)	243,955 (48.69)	<0.001
Race			<0.001
White	1,105,427 (69.42)	334,684 (68.41)	
Black	281,194 (17.66)	89,248 (18.24)	
Hispanic	124,957 (7.85)	39,938 (8.16)	
Asian/Pacific Islander	34,510 (2.17)	11,129 (2.27)	
Native/Other	8282 (0.52)	2678 (0.55)	
Unknown	37,991 (2.39)	11,588 (2.37)	
Hypertension	1,446,019 (88.60)	445,004 (88.81)	<0.001
Diabetes Mellitus	769,489 (47.15)	236,340 (47.17)	0.839
Hyperlipidemia	872,714 (53.47)	265,291 (52.94)	<0.001
Hyperthyroidism	13,058 (0.8)	3935 (0.69)	0.302
Hypothyroidism	295,618 (18.11)	90,679 (18.1)	0.784
Peripheral artery disease	111,768 (6.85)	32,417 (6.47)	<0.001
Coronary artery disease	958,810 (58.75)	297,266 (59.32)	<0.001
Atrial fibrillation/flutter	786,152 (48.17)	235,616 (47.02)	<0.001
COPD	594,480 (36.43)	187,657 (37.45)	<0.001
Obesity	414,872 (25.42)	123,153 (24.58)	<0.001
VT/VF	113,923 (6.98)	34,536 (6.89)	0.032
VTE	57,068 (3.50)	17,660 (3.52)	0.353
Stroke	63,208 (3.87)	19,434 (3.88)	0.862
CKD Stage 1–5 (No dialysis)	536,337 (32.86)	164,043 (32.74)	0.097
ESRD or dialysis	111,800 (6.85)	33,706 (6.73)	0.001

**Table 2:** Baseline Demographics of Patients Admitted with Acute Decompensated Heart Failure on Weekday vs. Weekend (after matching).

Variable	Weekday (*n* = 489,204)	Weekend (*n* = 489,204)	*p*-Value
Age (years)	Mean = 71.85, SD = 13.98	Mean = 71.69, SD = 14.12	0.34
Co-morbidities/Past medical history	(*n*, %)	(*n*, %)	
Female	239,174 (48.89)	238,424 (48.74)	0.129
Hypertension	436,579 (89.24)	435,003 (88.92)	0.054
Diabetes Mellitus	231,354 (47.29)	230,592 (47.21)	0.416
Hyperlipidemia	259,896 (53.13)	259,341 (53.01)	0.261
Hyperthyroidism	3885 (0.79)	3857 (0.79)	0.749
Hypothyroidism	88,475 (18.09)	88,520 (18.09)	0.906
Peripheral artery disease	31,015 (6.34)	31,553(6.45)	0.062
Coronary artery disease	290,619 (59.41)	290,155 (59.31)	0.34
Atrial fibrillation/Atrial flutter	230,617 (47.14)	230,154 (47.05)	0.348
COPD	183,229 (37.45)	183,539 (37.52)	0.517
Obesity	119,277 (24.38)	120,355 (24.6)	0.17
Ventricular Tachycardia/Ventricular Fibrillation	31,163 (6.37)	33,655 (6.88)	0.089
Venous Thromboembolism	14,854 (3.04)	17,178 (3.51)	0.354
Stroke	16,638 (3.4)	18,885 (3.86)	0.139
CKD Stage 1–5 (No dialysis)	160,685 (32.85)	160,285 (32.76)	0.389
ESRD or dialysis	32,134 (6.57)	33,046 (6.76)	0.73
Race			0.068
White	337,738 (69.04)	334,644 (68.41)	
Black	88,455 (18.08)	89,235 (18.24)	
Hispanic	39,054 (7.98)	39,934 (8.16)	
Asian/Pacific Islander	10,485 (2.14)	11,127 (2.27)	
Native/Other	2224 (0.45)	2678 (0.55)	
Unknown	11,248 (2.3)	11,586 (2.37)	

**Table 3:** Medical and Non-medical Outcomes of Patients Admitted with Acute Decompensated Heart Failure on a Weekday and a Weekend.

Variables	Weekday (*n* = 489,204)	Weekend (*n* = 489,204)	*p*-Value
Medical Outcomes	(*n*, %)	(*n*, %)	
ARF	105,603 (21.59)	128,295 (26.23)	<0.001
ARF with the use of mechanical ventilation	16,206 (3.31)	16,455 (3.36)	0.161
Hemodialysis initiation	29,750 (6.08)	29,621 (6.05)	0.585
AKI	177,900 (36.37)	186,732 (38.17)	<0.001
Cardiogenic shock during the stay	17,987 (3.68)	19,255 (3.93)	<0.001
In-hospital death	24,546 (5.02)	26,645 (5.45)	<0.001
Cardiac arrest	7822 (1.6)	8980 (1.84)	<0.001
Use of MCS			<0.001
None	483,005 (98.73)	483,470 (98.83)	
IABP	4175 (0.85)	3668 (0.75)	
Impella	1813 (0.37)	1711 (0.35)	
ECMO	211 (0.04)	355 (0.07)	
Non-medical Outcomes			
Length of Stay (days)	Mean = 6.89, SD = 7.6	Mean = 6.77, SD = 7.75	0.07
Total Hospital Charges	Mean = $78,913, SD = $141,298	Mean = $82,017, SD = $149,492	<0.001
Discharge Status	207,809 (42.51)	204,443 (41.81)	0.137
Home	15,826 (3.24)	15,342 (3.14)	
Short-term Hospital	120,637 (24.68)	120,620 (24.67)	
Another Type of Facility	114,385 (23.4)	114,863 (23.49)	
Home Health Care	5538 (1.13)	7030 (1.44)	
AMA	24,546 (5.02)	26,645 (5.45)	
Died in Hospital	85 (0.02)	64 (0.01)	

**Table 4:** Multivariate Logistic Regression for Patients Admitted with Acute Decompensated Heart Failure on a Weekend vs. a Weekday.

Variables	Adjusted Odds Ratio (95% CI)	*p*-Value
Inpatient death	1.07 (1.05–1.09)	<0.001
Cardiac arrest	1.1 (1.06–1.13)	<0.001
HD initiation	0.91 (0.89–1.01)	0.06
Use of MCS	0.87 (0.84–0.9)	<0.001
AKI	1.07 (1.06–1.08)	<0.001
ARFARF	1.28 (1.27–1.3)	<0.001
ARF Requiring Mechanical Ventilation	1.02 (0.99–1.04)	0.054

## Data Availability

This study utilized de-identified publicly available data through the Healthcare Cost and Utilization Project (HCUP) website, https://hcup-us.ahrq.gov.

## Supplementary Materials

The following supporting information can be downloaded at: https://www.mdpi.com/article/10.3390/jcm15062097/s1, Table S1: ICD-10 Codes Used for Variables and Data Extraction.
