# Supplementary material for: Increased Mortality and Complication Rates in Weekend Admissions for Acute Decompensated Heart Failure: A Five-Year National Study
Source: J Clin Med. 2026 Mar 10;15(6):2097. doi: 10.3390/jcm15062097 (PMC13026610; doi:10.3390/jcm15062097)
Supplement: Supplementary file 1 [file jcm-15-02097-s001.zip › jcm-4187953-supplementary.pdf]

Table S1. ICD-10 Codes Used for Variables and Data Extraction

Co-morbidities	ICD-10 Codes
**Atrial Fibrillation/flutter**	I48.0, I48.1, I48.2, I48.3, I48.4, I48.91, I48.92
**End-Stage Renal Disease (ESRD)**	N18.6
**Ischemic Stroke**	I63.0, I63.1, I63.2, I63.3, I63.4, I63.5, I63.6, I63.8, I63.9
**Hemorrhagic Stroke**	I61.0, I61.1, I61.2, I61.3, I61.4, I61.5, I61.6, I61.8, I61.9, I62.0, I62.1, I62.9
**Transient Ischemic Attack (TIA)**	G45.0, G45.1, G45.2, G45.3, G45.4, G45.8, G45.9
**Diabetes Mellitus**	E08.x, E09.x, E10.x, E11.x, E13.x
**Hypertension**	I10, I11.x, I12.x, I13.x, I15.x
**Dyslipidemia**	E78.0, E78.1, E78.2, E78.3, E78.4, E78.5, E78.6, E78.8, E78.9
**Coronary Artery Disease**	I20.x, I21.x, I22.x, I23.x, I24.x, I25.x
**Peripheral Vascular Disease**	I70.x, I73.9
**Chronic Obstructive Pulmonary Disease**	J43.x, J44.x
**Obesity**	E66.01
**Venous Thromboembolism (DVT/PE)**	I26.x, I80.x, I82.4, I82.5, I82.6, I82.7, I82.8, I82.9
**Hyperthyroidism**	E05.0, E05.1, E05.2, E05.3, E05.4, E05.5, E05.8, E05.9
**Hypothyroidism**	E03.0–E03.5, E03.8, E03.9
**Ventricular Tachycardia/Ventricular Fibrillation**	I47.2, I49.01
**Acute Respiratory Failure**	J96.00, J96.01, J96.02, J96.09
**Use of Mechanical Ventilation**	5A1935Z, 5A1945Z, 5A1955Z
**Acute Kidney Injury**	N17.0, N17.1, N17.2, N17.8, N17.9
**Initiation of Hemodialysis**	5A1D60Z, 5A1D61Z, 5A1D62Z, 5A1D70Z–5A1D72Z
**Cardiogenic Shock**	R57.0

**Use of Mechanical Circulatory Support (Intra-Aortic Balloon Pump)**	5A02210
**Use of Mechanical Circulatory Support (Impella)**	02HA3RJ, 02HA3RZ, 5A0221D
**Use of Mechanical Circulatory Support (ECMO)**	5A1522F, 5A1522G
**Cardiac Arrest**	I46.0, I46.2, I46.9
